# Supplementary material for: The septin cytoskeleton is required for plasma membrane repair
Source: EMBO Rep. 2024 Jul 5;25(9):11. doi: 10.1038/s44319-024-00195-6 (PMC11387490; doi:10.1038/s44319-024-00195-6)
Supplement: Supplementary file 1 — Table EV1 [file 44319_2024_195_MOESM1_ESM.docx]

| GENES | Estimate | st. dev. | P values |  | GENES | Estimate | st. dev. | P values |
| --- | --- | --- | --- | --- | --- | --- | --- | --- |
| Synaptotagmins | | | |  | Coatomer | | | |
| *SYT10* | 1.2844 | 0.1879 | <.0001 |  | *PREB* | 0.8879 | 0.2663 | 0.002 |
| *SYT12* | 0.6103 | 0.2156 | 0.0076 |  | *SAR1B* | 0.8366 | 0.2742 | 0.0043 |
| *SYT14* | 1.0435 | 0.1879 | <.0001 |  | *SEC23A* | 0.8038 | 0.2491 | 0.0027 |
| *SYT4* | 0.9706 | 0.3385 | 0.0069 |  | *SEC24B* | 0.5802 | 0.2491 | 0.0255 |
| SNARE | | | |  | RAB | | | |
| *SNAP25* | 1.0636 | 0.2779 | 0.0005 |  | *RAB11A* | 0.6593 | 0.2037 | 0.0026 |
| *SNAP29* | 0.6257 | 0.2663 | 0.0244 |  | *RAB27A* | 0.6589 | 0.2037 | 0.0026 |
| *VAMP3* | 0.5795 | 0.2663 | 0.0362 |  | *RAB27B* | 0.7774 | 0.2037 | 0.0005 |
| *VAMP8* | 0.9043 | 0.2836 | 0.003 |  | *RAB6C* | 0.7302 | 0.213 | 0.0015 |
| *STX11* | 0.8029 | 0.2836 | 0.0076 |  | *RAB7A* | 0.5283 | 0.2037 | 0.0137 |
| *STX16* | 0.9561 | 0.2836 | 0.0018 |  | ***RAB7L1*** | 0.4598 | 0.2037 | 0.0302 |
| *STX19* | 1.0977 | 0.1879 | <.0001 |  | *RABL3* | 1.2823 | 0.1879 | <.0001 |
| *STX1A* | 0.7996 | 0.2779 | 0.0067 |  | *RGS6* | 0.8321 | 0.2663 | 0.0035 |
| *STX4* | 0.9928 | 0.2779 | 0.001 |  | ***RAB2A*** | *-0.3347* | *0.1263* | *0.0119* |
| *STX8* | 0.7836 | 0.2663 | 0.0057 |  | ***RAB40B*** | *-0.9774* | *0.2851* | *0.0015* |
| *STXBP1* | 0.786 | 0.2779 | 0.0076 |  | ***RAB4A*** | *-0.397* | *0.1263* | *0.0033* |
| *STXBP4* | 0.9842 | 0.1879 | <.0001 |  | ***RAB7B*** | *-0.6466* | *0.1889* | *0.0016* |
| Calpains | | | |  | Intracellular trafficking | | | |
| *CAPN1* | 0.5569 | 0.2466 | 0.03 |  | *VPS13B* | 0.9997 | 0.1879 | <.0001 |
| Annexins | | | |  | ***LMAN1*** | *-1.2104* | *0.2409* | *<.0001* |
| *ANXA2* | 0.59 | 0.2642 | 0.0319 |  | Endocytosis | | | |
| *ANXA7* | 0.7559 | 0.2642 | 0.007 |  | ***CLTA*** | *-0.5487* | *0.2026* | *0.0103* |
| *ANXA11* | 0.5525 | 0.2642 | 0.0436 |  | ***CLTB*** | *-0.6981* | *0.2026* | *0.0015* |
| ESCRT-I, -III | | | |  | ***DNM2*** | *-1.0475* | *0.2409* | *0.0001* |
| *UBAP1* | 0.6887 | 0.2466 | 0.0083 |  | ***AP2S1*** | *-0.5095* | *0.2026* | *0.0165* |
| *CHMP2A* | 0.7421 | 0.1991 | 0.0007 |  | Exocyst complex | | | |
| Organelle biogenesis / lysosomes | | | |  | *EXOC1* | 0.3266 | 0.1568 | 0.0444 |
| *PLIN3* | 0.761 | 0.2491 | 0.0042 |  | ***EXOC7*** | *-0.6362* | *0.1702* | *0.0006* |
| *HPS3* | 0.6083 | 0.2156 | 0.0077 |  | Guanine Exchange Factors (GEF) | | | |
| *SMPD1* | 0.8942 | 0.2532 | 0.0012 |  | *IQSEC1* | 0.7637 | 0.2663 | 0.0069 |
| Adaptor Complexes | | | |  |  |  |  |  |
| *AP3D1* | 0.7959 | 0.23 | 0.0014 |  | *GBF1* | 0.9967 | 0.2836 | 0.0012 |
| *AP3M2* | 0.6353 | 0.2491 | 0.0151 |  | Cytoskeletal regulation | | | |
| *AP3S2* | 0.8485 | 0.2491 | 0.0016 |  | *SEPT7*  *ABI1* | 0.5257  0.5397 | 0.2466  0.1702 | 0.0399  0.0031 |
| *AP4B1* | 0.7971 | 0.2491 | 0.0029 |  |  |  |  |  |

**Table EV1. Genes controlling plasma membrane integrity**. The screen was conducted with HeLa cells and targeted 245 genes with a cocktail of three non-overlapping siRNAs (see Data Set EV1). The assay measured TO-PRO-3 fluorescence intensity as a readout for plasma membrane integrity of cells incubated with or without 0.5 nM LLO for 30 min. Data are the statistical analyses comparing targeting siRNA-treated cells to cells treated with control siRNA. Silencing 47 genes led to a significant decrease in plasma membrane integrity and silencing 10 genes (in bold, negative values) significantly improved plasma membrane integrity. Gene names are according to the HUGO Gene Nomenclature (HGNC, [genenames.org)](https://www.genenames.org/) and GeneCards (Genecards.org).
