# Supplementary figures and images for: The septin cytoskeleton is required for plasma membrane repair
Source: EMBO Rep. 2024 Jul 5;25(9):11. doi: 10.1038/s44319-024-00195-6 (PMC11387490; doi:10.1038/s44319-024-00195-6)

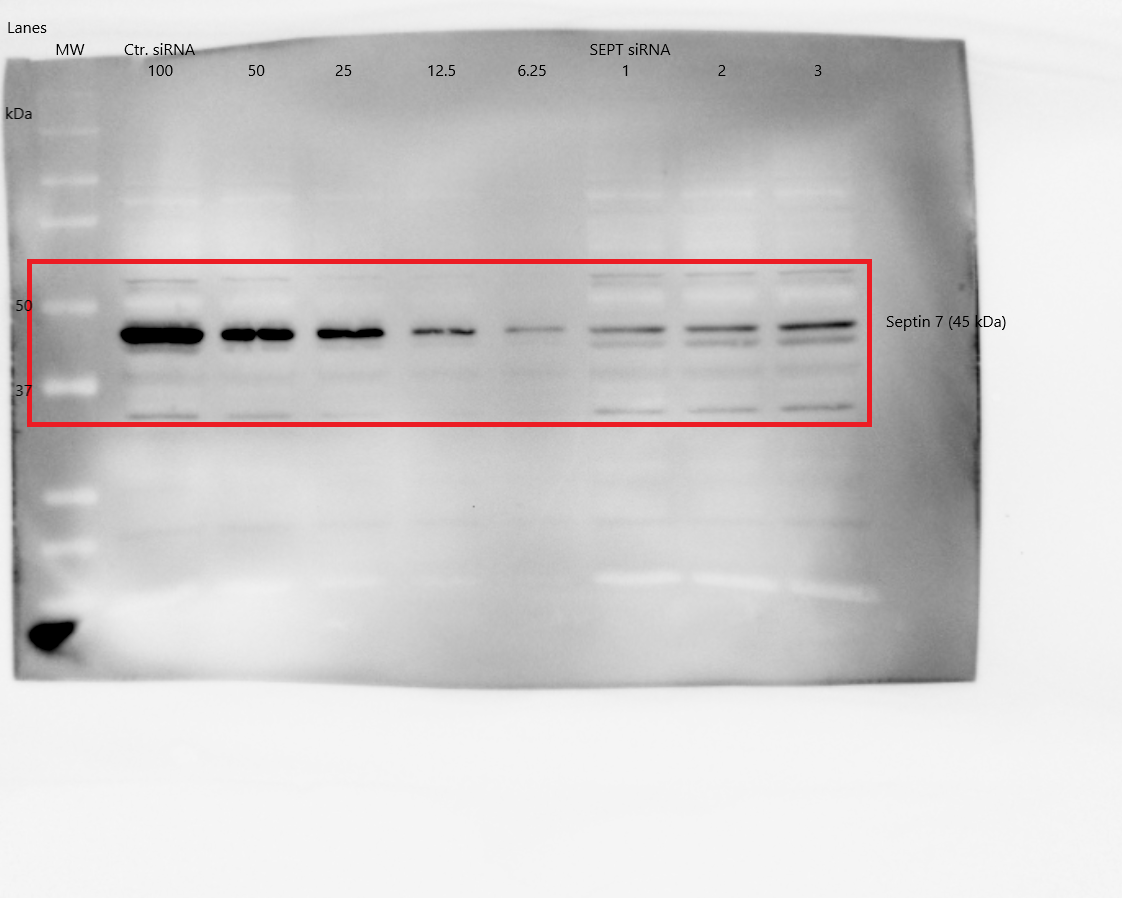

Supplement: Supplementary file 7 — Source data Fig. 1 [file 44319_2024_195_MOESM7_ESM.zip › Figure 1/Fig1A/1A 230221 Septin 7 KD annotated.tif]

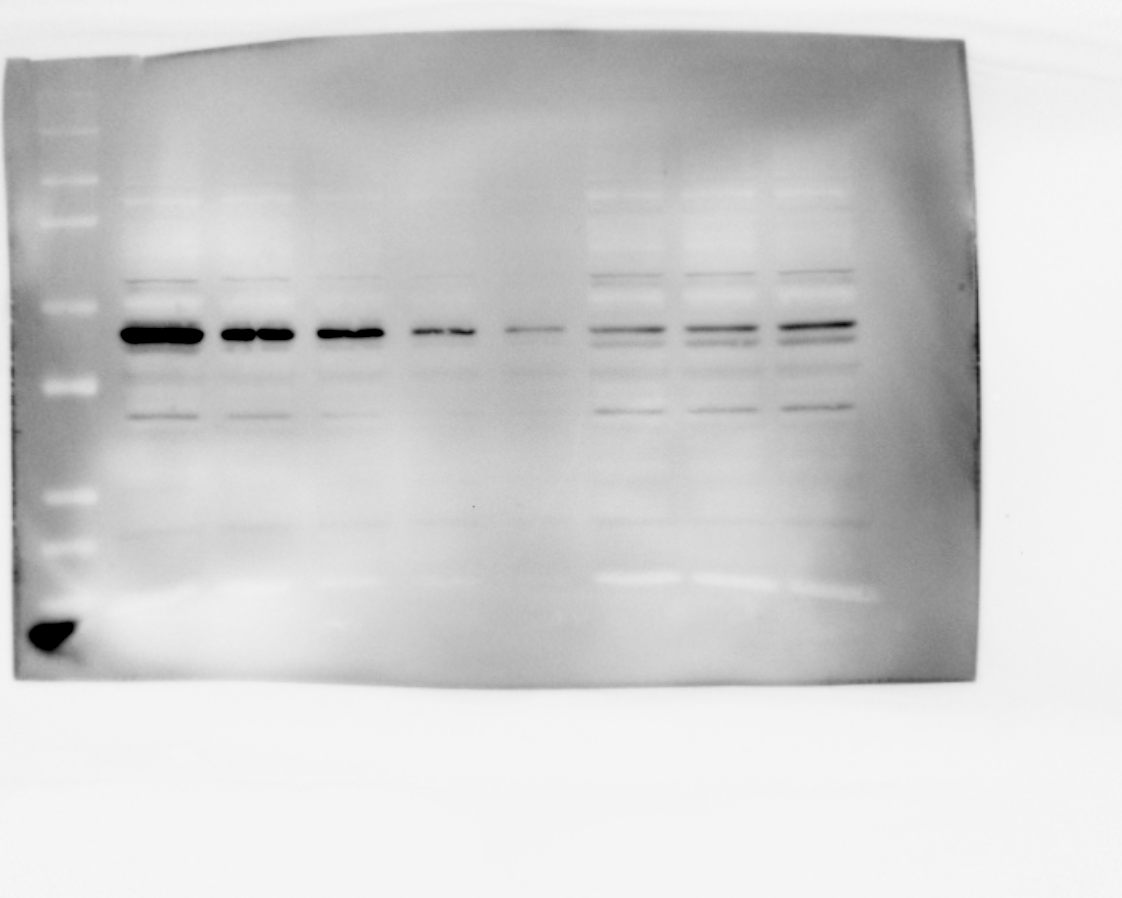

Supplement: Supplementary file 7 — Source data Fig. 1 [file 44319_2024_195_MOESM7_ESM.zip › Figure 1/Fig1A/1A 230221 Septin 7 KD.tif]

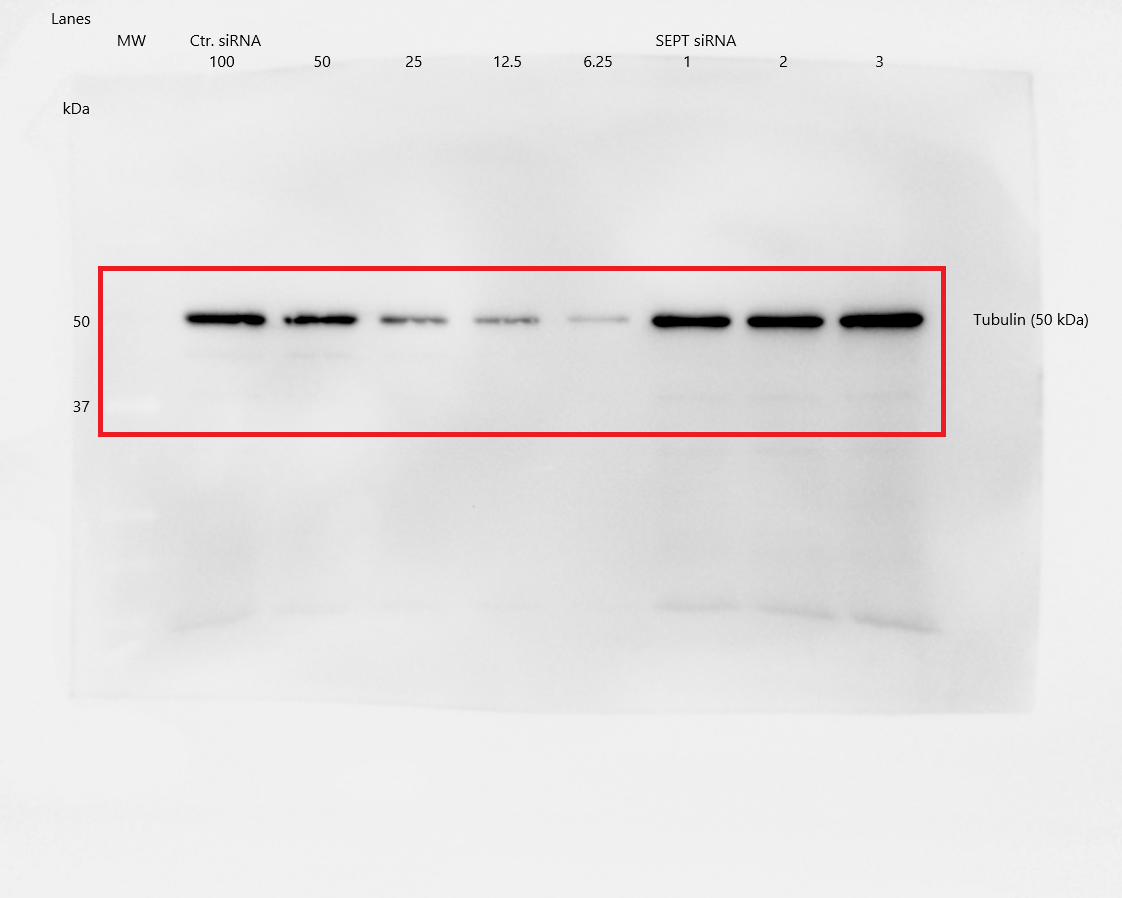

Supplement: Supplementary file 7 — Source data Fig. 1 [file 44319_2024_195_MOESM7_ESM.zip › Figure 1/Fig1A/1A 230222 Septin 7 KD - tubulin annotated.tif]

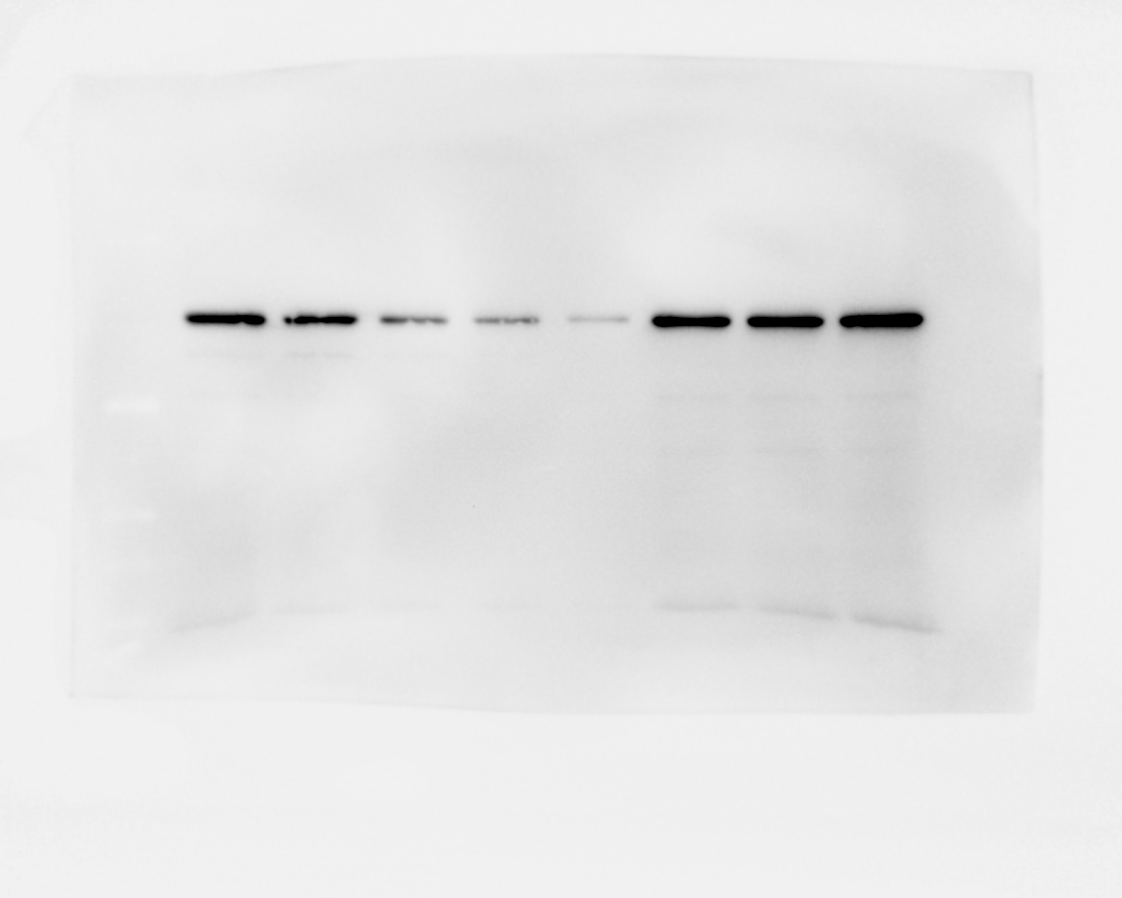

Supplement: Supplementary file 7 — Source data Fig. 1 [file 44319_2024_195_MOESM7_ESM.zip › Figure 1/Fig1A/1A 230222 Septin 7 KD - tubulin.tif]

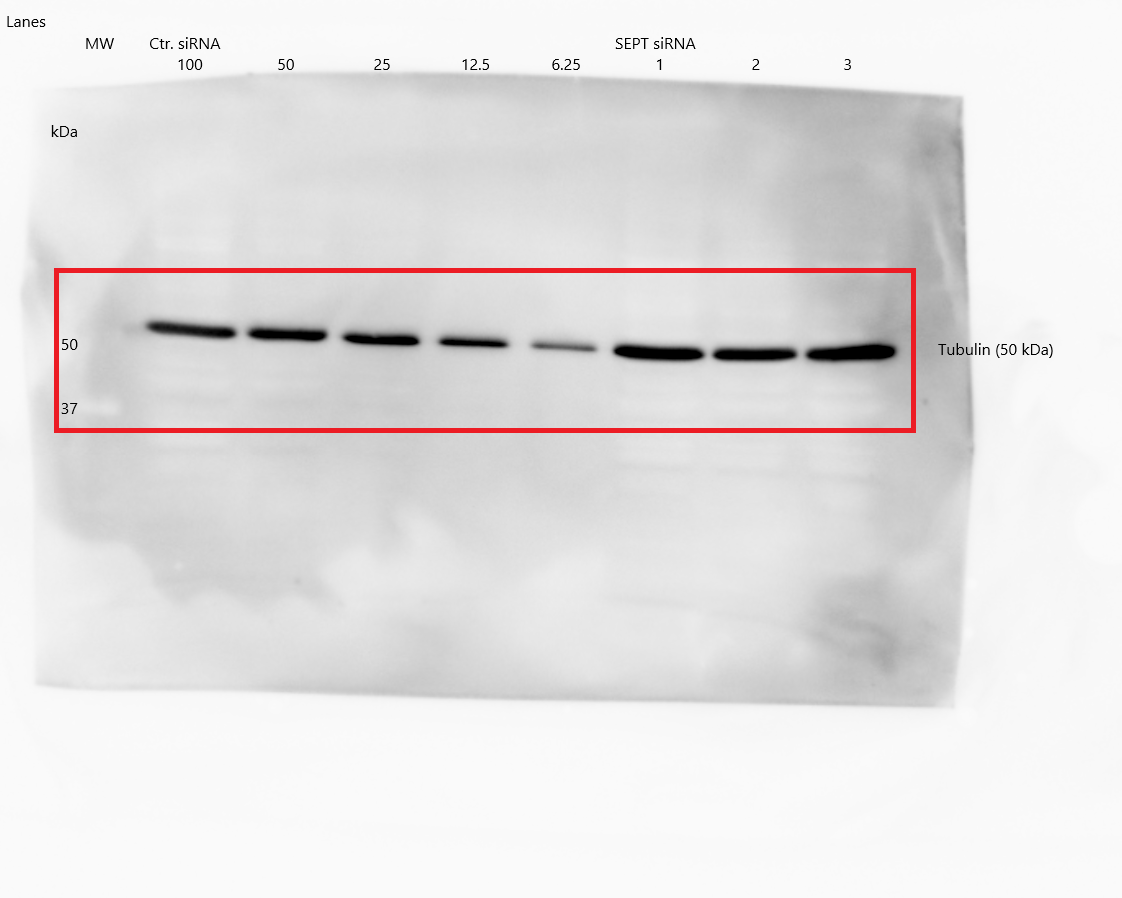

Supplement: Supplementary file 7 — Source data Fig. 1 [file 44319_2024_195_MOESM7_ESM.zip › Figure 1/Fig1A/1A 230321 Septin 6 KD - tubulin annotated.tif]

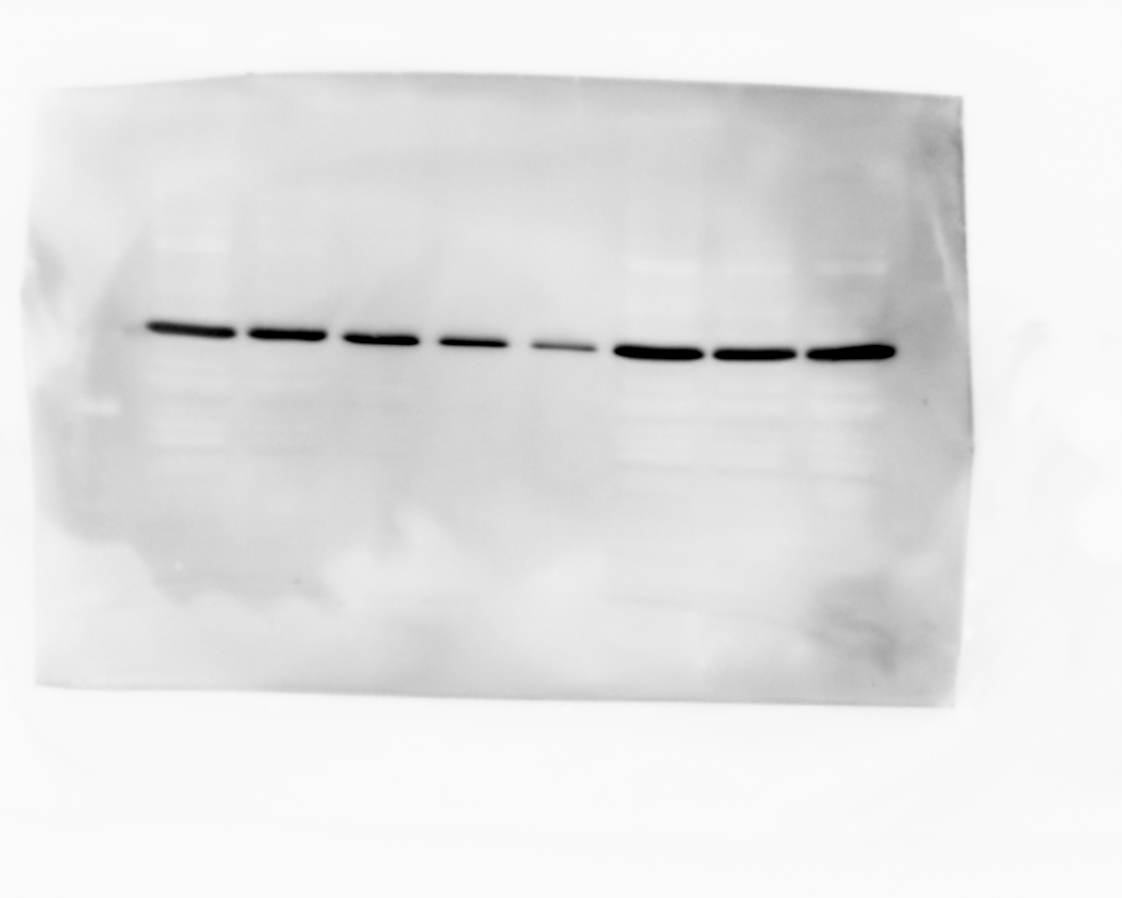

Supplement: Supplementary file 7 — Source data Fig. 1 [file 44319_2024_195_MOESM7_ESM.zip › Figure 1/Fig1A/1A 230321 Septin 6 KD - tubulin.tif]

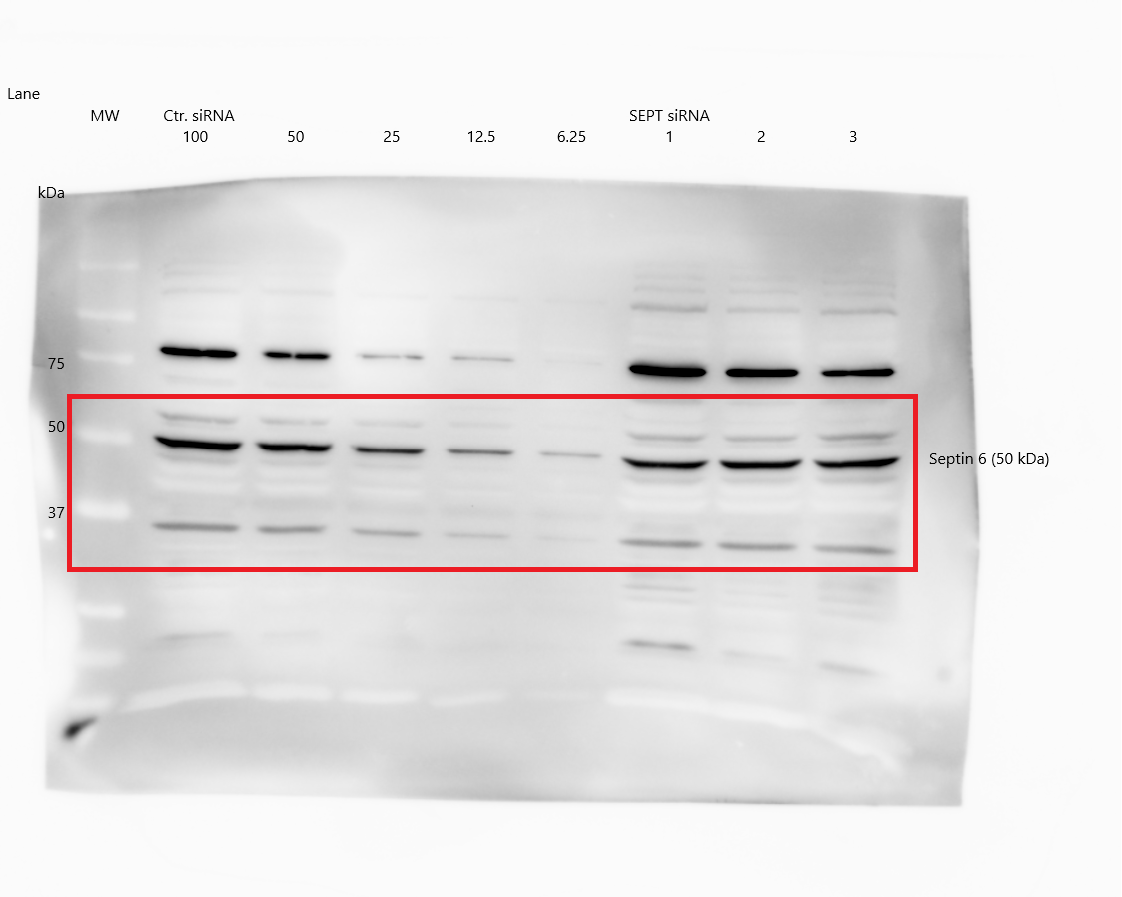

Supplement: Supplementary file 7 — Source data Fig. 1 [file 44319_2024_195_MOESM7_ESM.zip › Figure 1/Fig1A/1A 230321 Septin 6 KD annotated.tif]

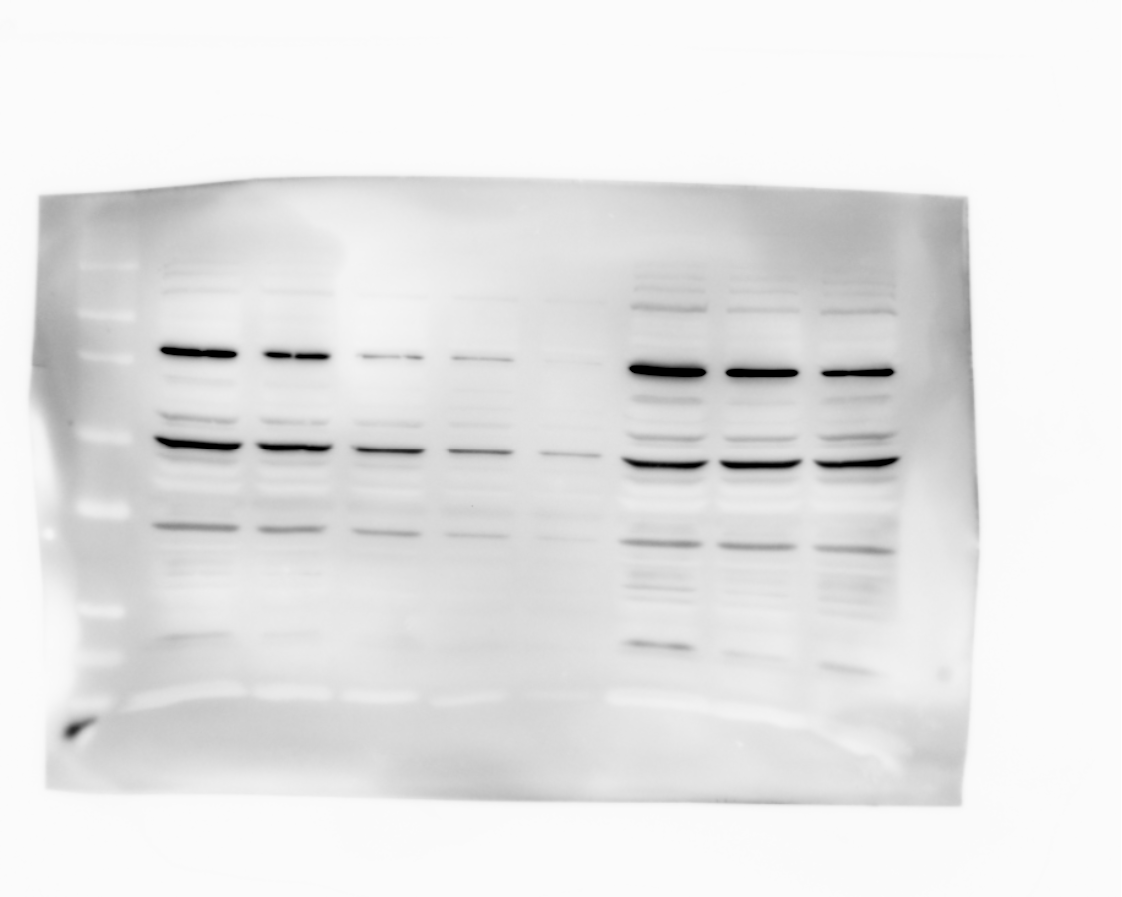

Supplement: Supplementary file 7 — Source data Fig. 1 [file 44319_2024_195_MOESM7_ESM.zip › Figure 1/Fig1A/1A 230321 Septin 6 KD.tif]

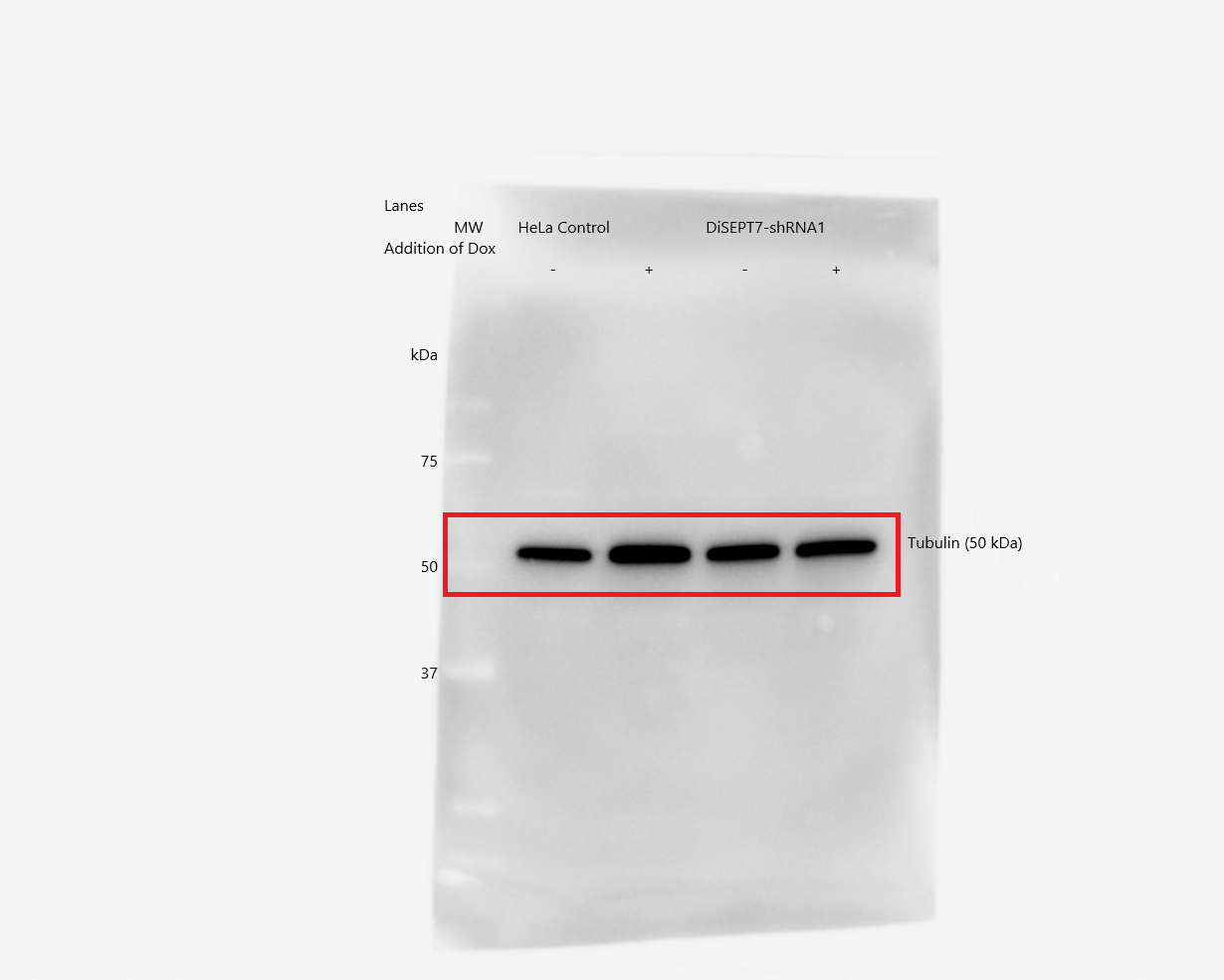

Supplement: Supplementary file 7 — Source data Fig. 1 [file 44319_2024_195_MOESM7_ESM.zip › Figure 1/Fig1E/1E 220923 Septin 7 shRNA KD - tubulin annotated.tif]

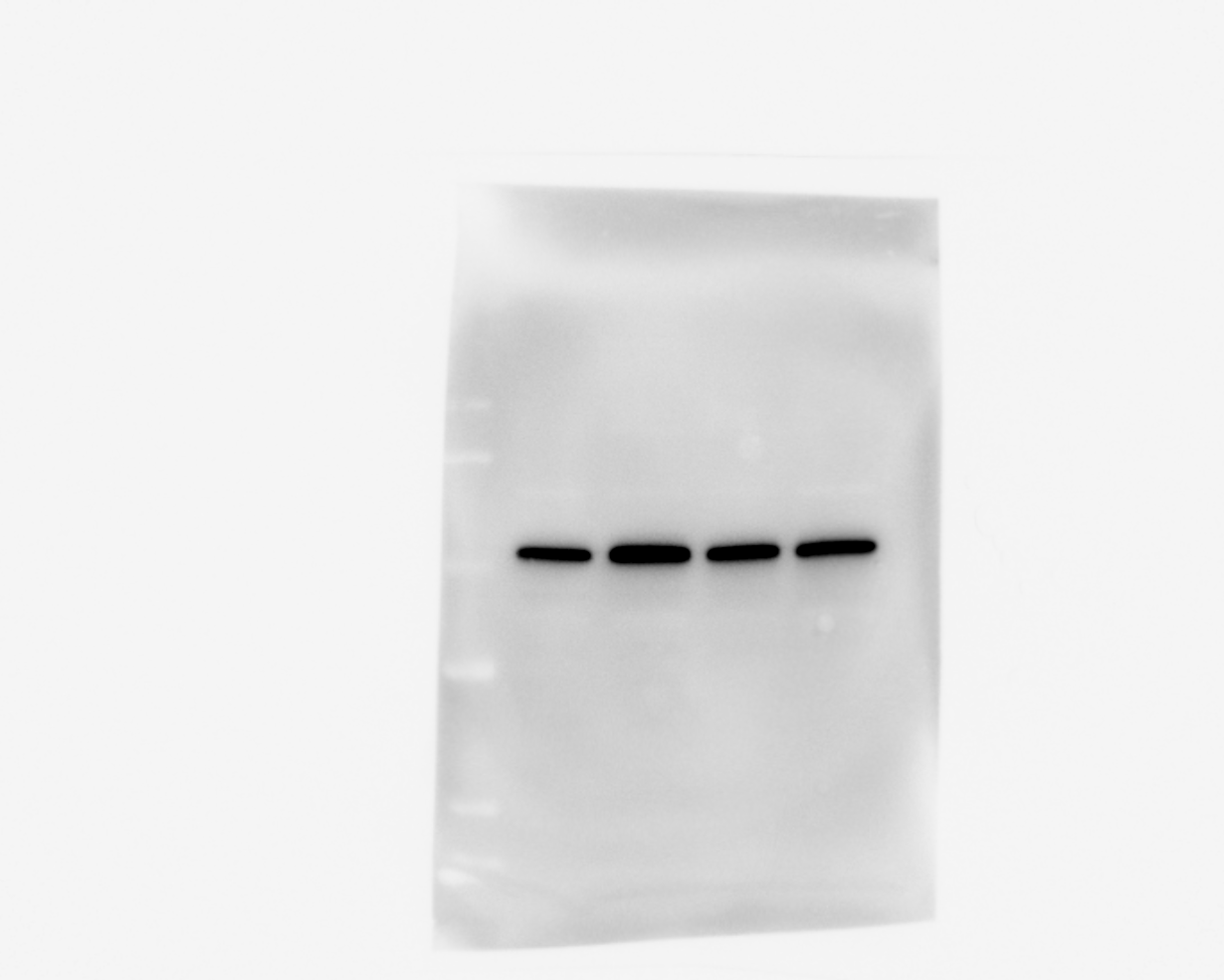

Supplement: Supplementary file 7 — Source data Fig. 1 [file 44319_2024_195_MOESM7_ESM.zip › Figure 1/Fig1E/1E 220923 Septin 7 shRNA KD - tubulin.tif]

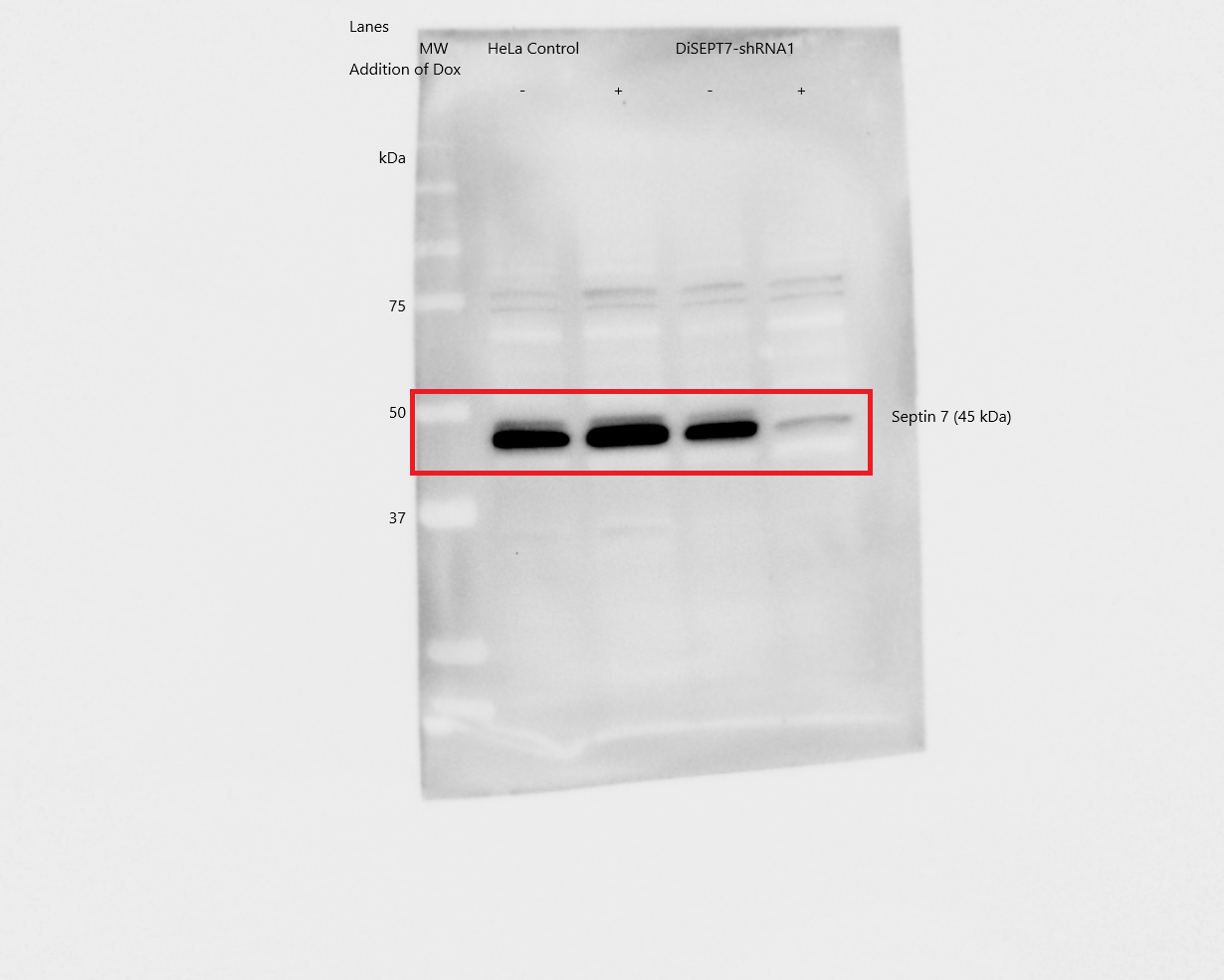

Supplement: Supplementary file 7 — Source data Fig. 1 [file 44319_2024_195_MOESM7_ESM.zip › Figure 1/Fig1E/1E 220923 Septin 7 shRNA KD annotated.tiff]

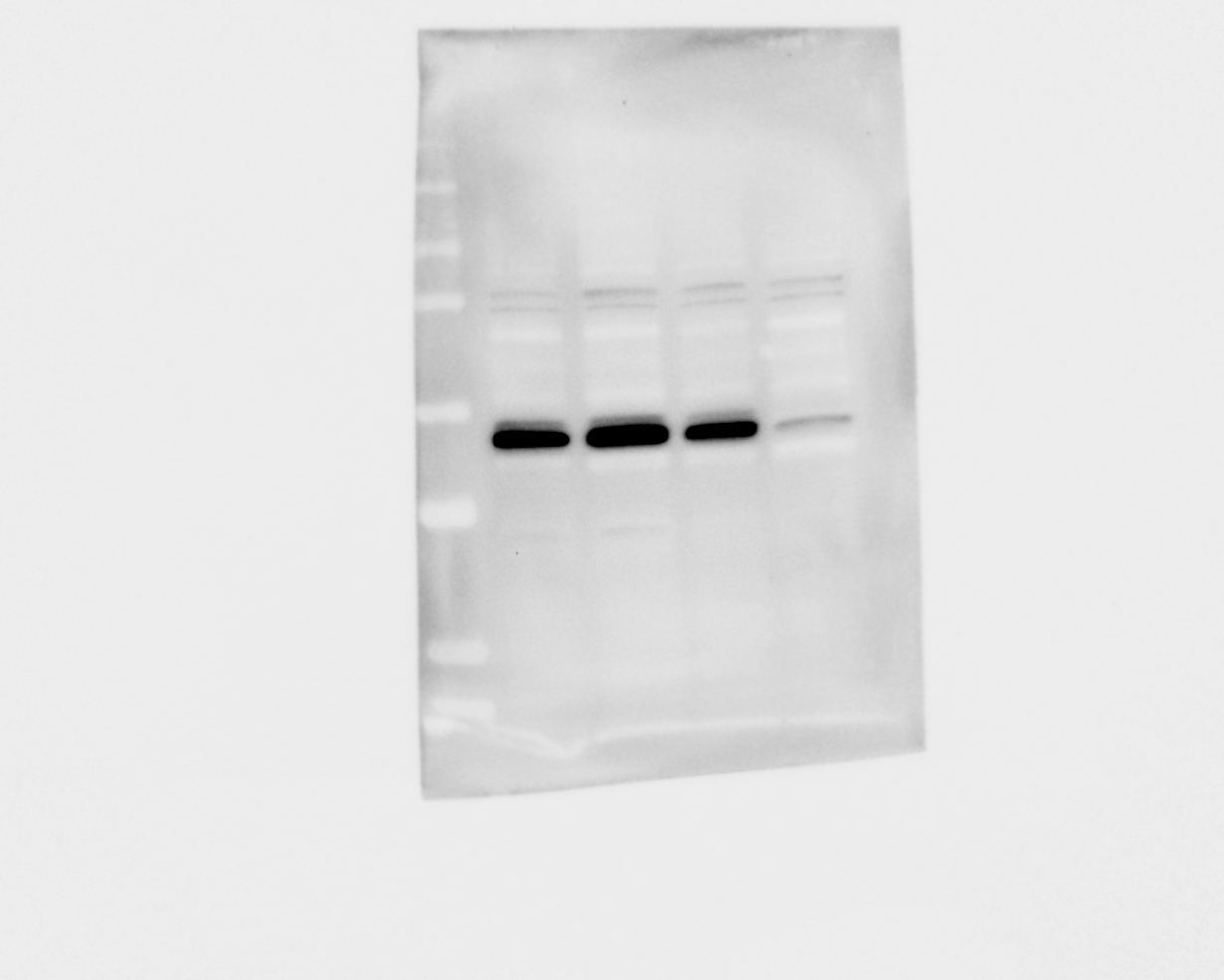

Supplement: Supplementary file 7 — Source data Fig. 1 [file 44319_2024_195_MOESM7_ESM.zip › Figure 1/Fig1E/1E 220923 Septin 7 shRNA KD.tif]

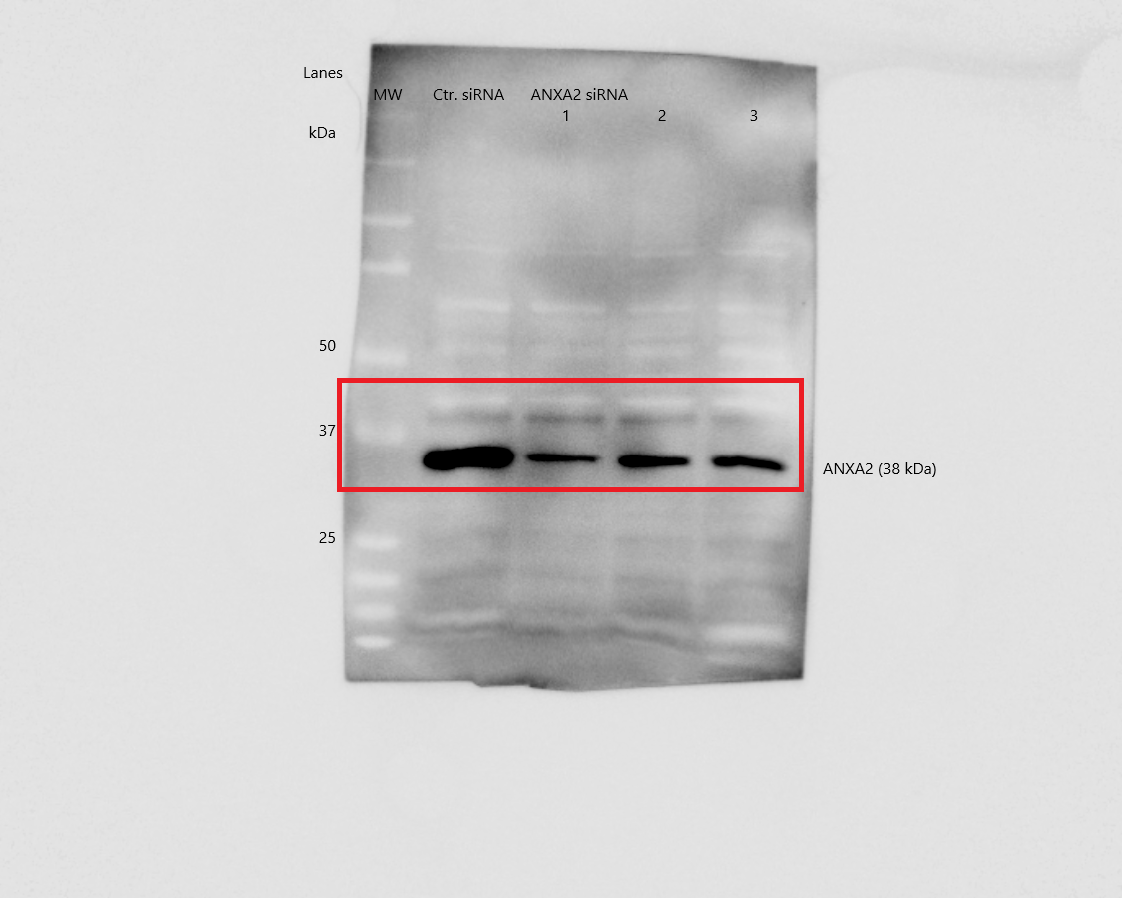

Supplement: Supplementary file 7 — Source data Fig. 1 [file 44319_2024_195_MOESM7_ESM.zip › Figure 1/Fig1I/1I 230531 ANXA2 KD annotated.tif]

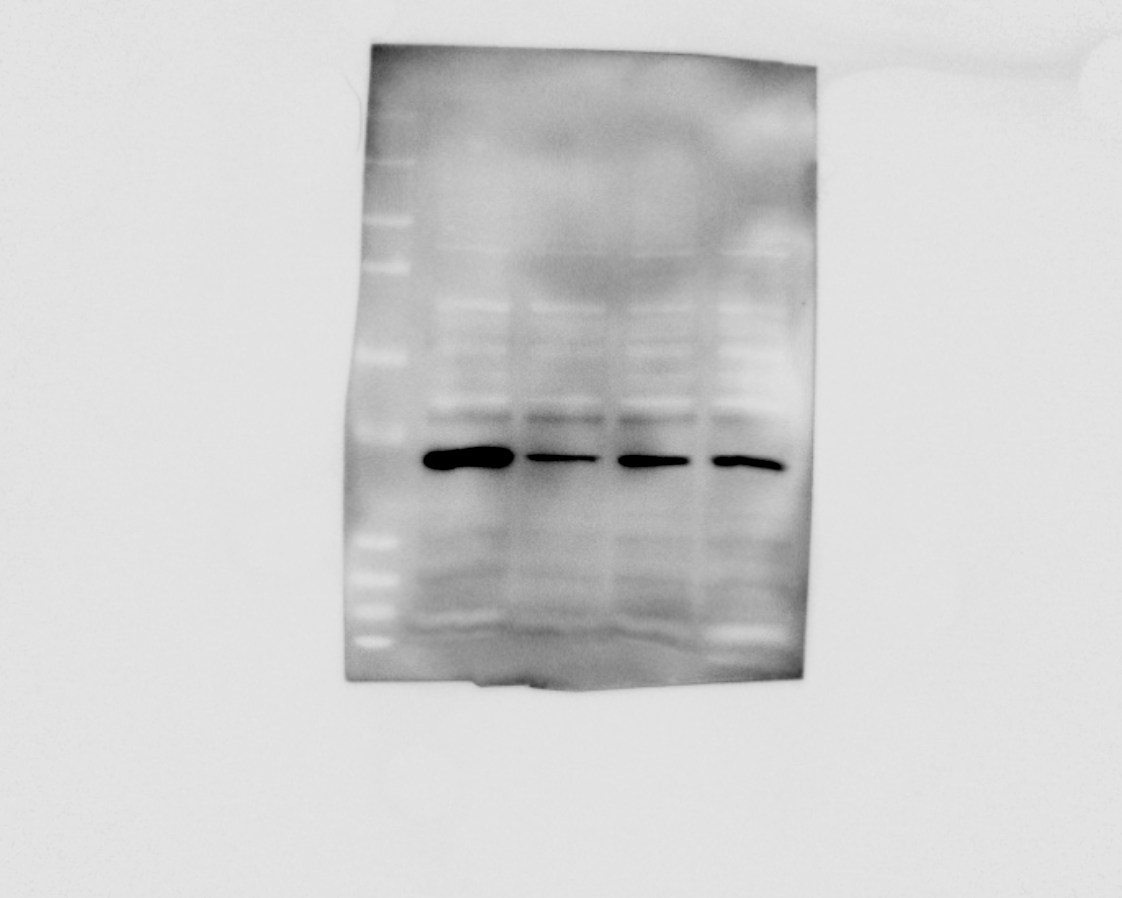

Supplement: Supplementary file 7 — Source data Fig. 1 [file 44319_2024_195_MOESM7_ESM.zip › Figure 1/Fig1I/1I 230531 ANXA2 KD.tif]

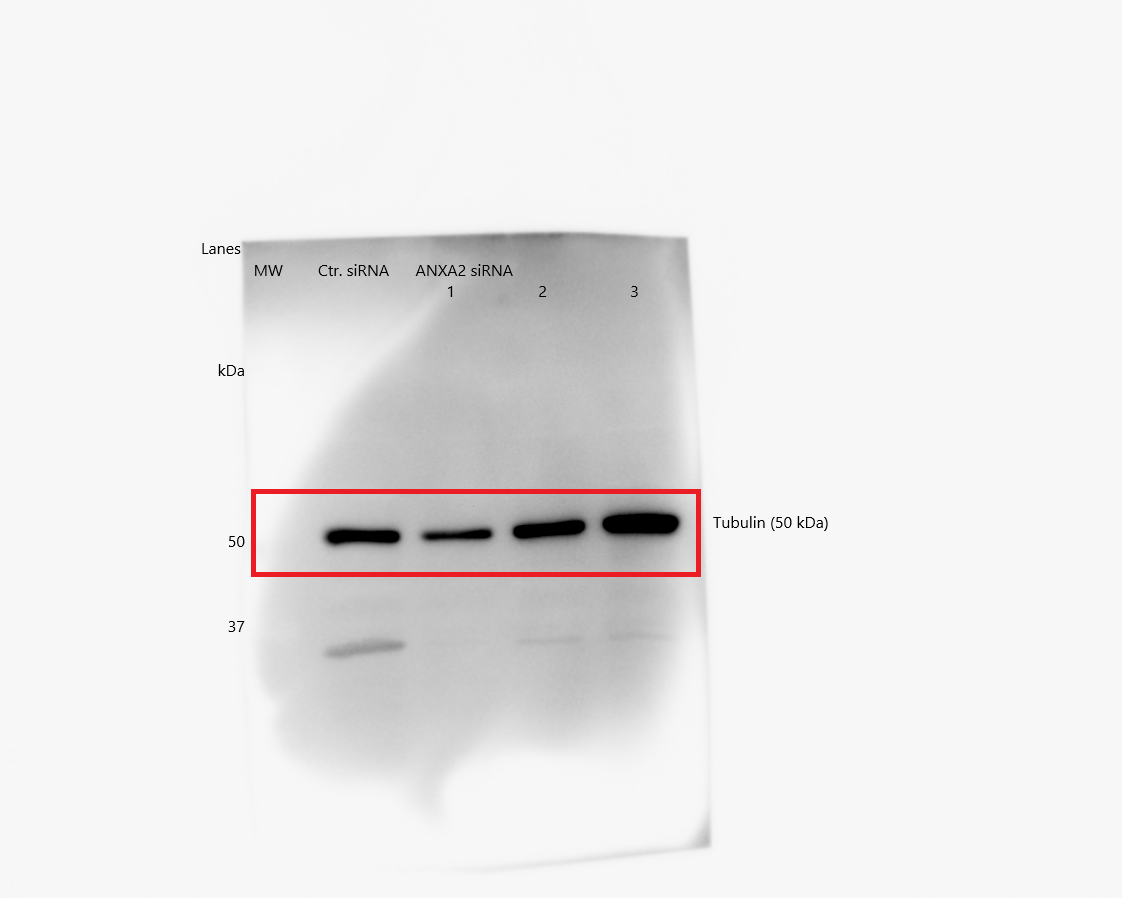

Supplement: Supplementary file 7 — Source data Fig. 1 [file 44319_2024_195_MOESM7_ESM.zip › Figure 1/Fig1I/1I 230601 ANXA2 KD tubulin annotated.tif]

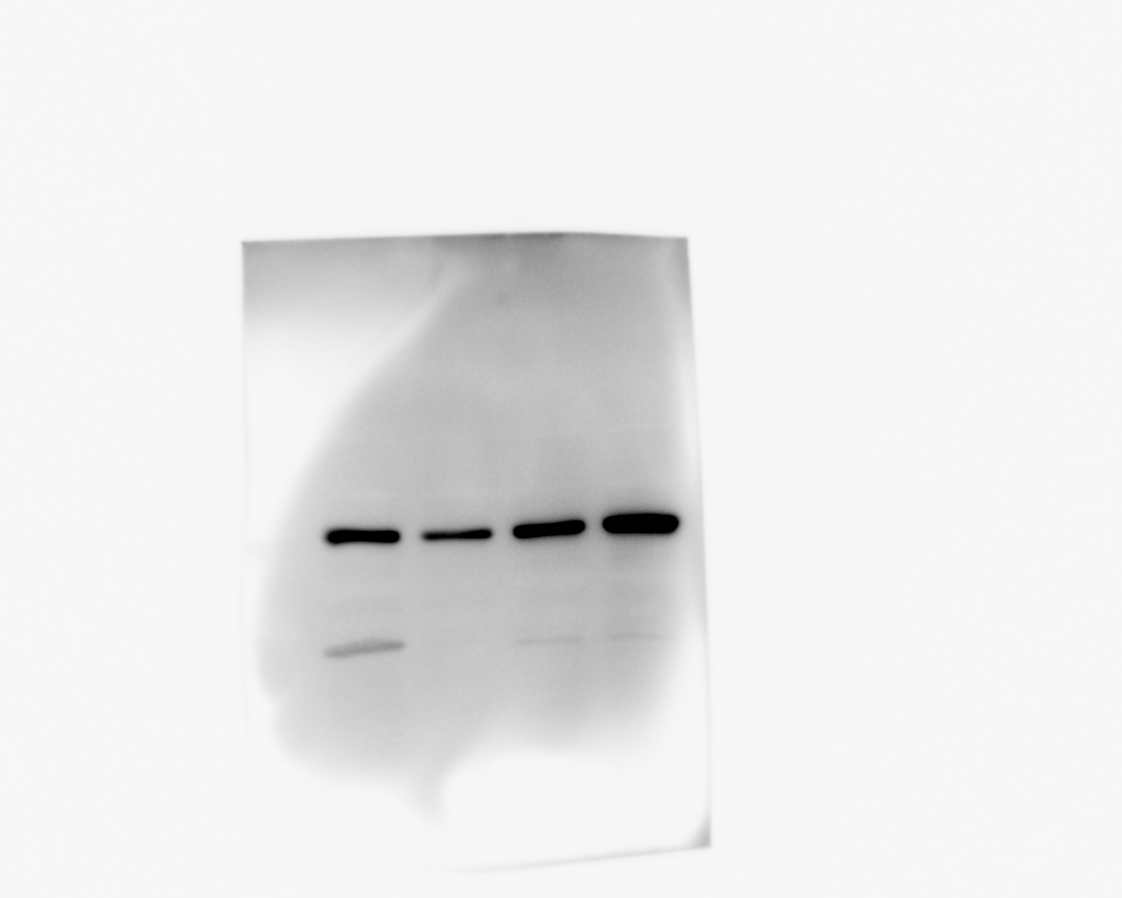

Supplement: Supplementary file 7 — Source data Fig. 1 [file 44319_2024_195_MOESM7_ESM.zip › Figure 1/Fig1I/1I 230601 ANXA2 KD tubulin.tif]
