# Supplementary material for: The septin cytoskeleton is required for plasma membrane repair
Source: EMBO Rep. 2024 Jul 5;25(9):11. doi: 10.1038/s44319-024-00195-6 (PMC11387490; doi:10.1038/s44319-024-00195-6)
Supplement: Supplementary file 12 — Expanded View Figures [file 44319_2024_195_MOESM12_ESM.pdf]

Expanded View Figures

Figure EV1. SEPT2 and SEPT9 silencing do not affect plasma membrane repair.

(**A**) HeLa cells were transfected with Ctr.-, SEPT2-, or SEPT9-siRNAs (Dataset EV1). After 72 h, cells were lysed and analyzed by SDS-PAGE and immunoblotting for septin and tubulin expression. Serial dilutions of control- (100% to 6.25%) and undiluted septin- (100%) siRNA treated cell lysates were loaded in the gel to facilitate the quantification of the KD efficiencies. Blots are representative of at least $N = 5$. KD efficiencies were >90%. (**A'**) HeLa cells were treated for 72 h with Ctr. or SEPT-siRNAs and were exposed to LLO (0.5 nM) for 30 min in M1 containing TO-PRO-3. Data are the average TO-PRO-3 fluorescence intensities expressed in arbitrary units (AI) ± SEM of at least $N = 3$ independent experiments at time point 30 min. (**B**) HeLa cells were exposed, or not, to 0.5 nM LLO in M1 or M2 for 5 min on ice to allow LLO to bind the plasma membrane. Cells were washed and warmed up to 37 °C for 5, 10, 15, 30 min, 2 h, and 24 h. In the last min of incubation, cells were exposed for 1 min to 100 μM propidium iodide. About 1300 cells were analyzed per experimental condition and data are expressed as the average nuclear Propidium Iodide fluorescence intensities expressed in arbitrary units (AI) ± SEM of at least $N = 3$ independent experiments. $N = 4$ independent experiments for the 5-30 min conditions. Data show that plasma membrane integrity is recovered fully between 30 min to 2 h, at the whole population level. (**C–F**) HeLa cells were transfected with Ctr.- or SEPT-siRNAs as in (**A**). Blots are representative of at least $N = 5$. (**E**) Comparing control siRNA-treated cells to SEPT7-siRNA-treated cells, SEPT6 expression was reduced by 44.8% ± 24.4; 70% ± 21.2; and 52.3% ± 19.6 for siRNA1, 2, and 3, respectively, SEPT2 expression was reduced by 59.6% ± 4.3; 46.4% ± 7.7; and 40.1% ± 6.9 for siRNA1, 2, and 3, respectively. SEPT 9 expression was reduced by at least 90% for all three siRNAs. KD of SEPT2, 6, and 9 did not appear to affect the expression of the other tested septins. (**G**) HeLa cells were transfected with non-targeting Ctr. siRNA, SEPT7-siRNA 1, or SEPT2-siRNA 2. Cells were incubated without LLO for 15 min in M1 (1.2 mM Ca^{2+}), fixed, permeabilized, and labeled with anti-SEPT9 primary Ab (and Alexa Fluor 568-conjugated secondary), F-actin (Alexa Fluor 488-conjugated phalloidin), and nuclei (DAPI). Z-stack images were acquired by widefield microscopy, denoised, deconvolved, and displayed as best-focus projection images. Scale bars: 10 μm. (**H**) The hemolytic assay showing that the presence of DOX at the indicated concentrations does not affect LLO pore formation ($N = 3$ independent experiments, Average ± SEM). Note that in the repair assay, DOX-treated cells were washed three times, so only traces of DOX were present in the medium during the repair assay. (**I**, **J**) HeLa cells were pre-treated with the indicated concentrations of FCF, or corresponding DMSO dilutions, or left untreated (Ctr.) for 16 h. Equivalent concentrations of FCF and DMSO were maintained in the buffer during the repair assay. Cells were exposed to LLO for 30 min in M1 or M2 supplemented with TO-PRO-3. Data are expressed as the average TO-PRO-3 fluorescence intensities in arbitrary unit of $N = 2$ to $N = 4$ (each with 4 to 8 technical replicates) ± SEM at each time point. (**K**) The hemolytic assay showed that FCF at the highest concentration does not affect LLO pore formation ($N = 3$ independent experiments, Average ± SEM). Data Information: (**A'**, **B**) Data were $\log_{10}$ transformed and analyzed using linear mixed-effects models. $^*P < 0.05$.

 © The Author(s)

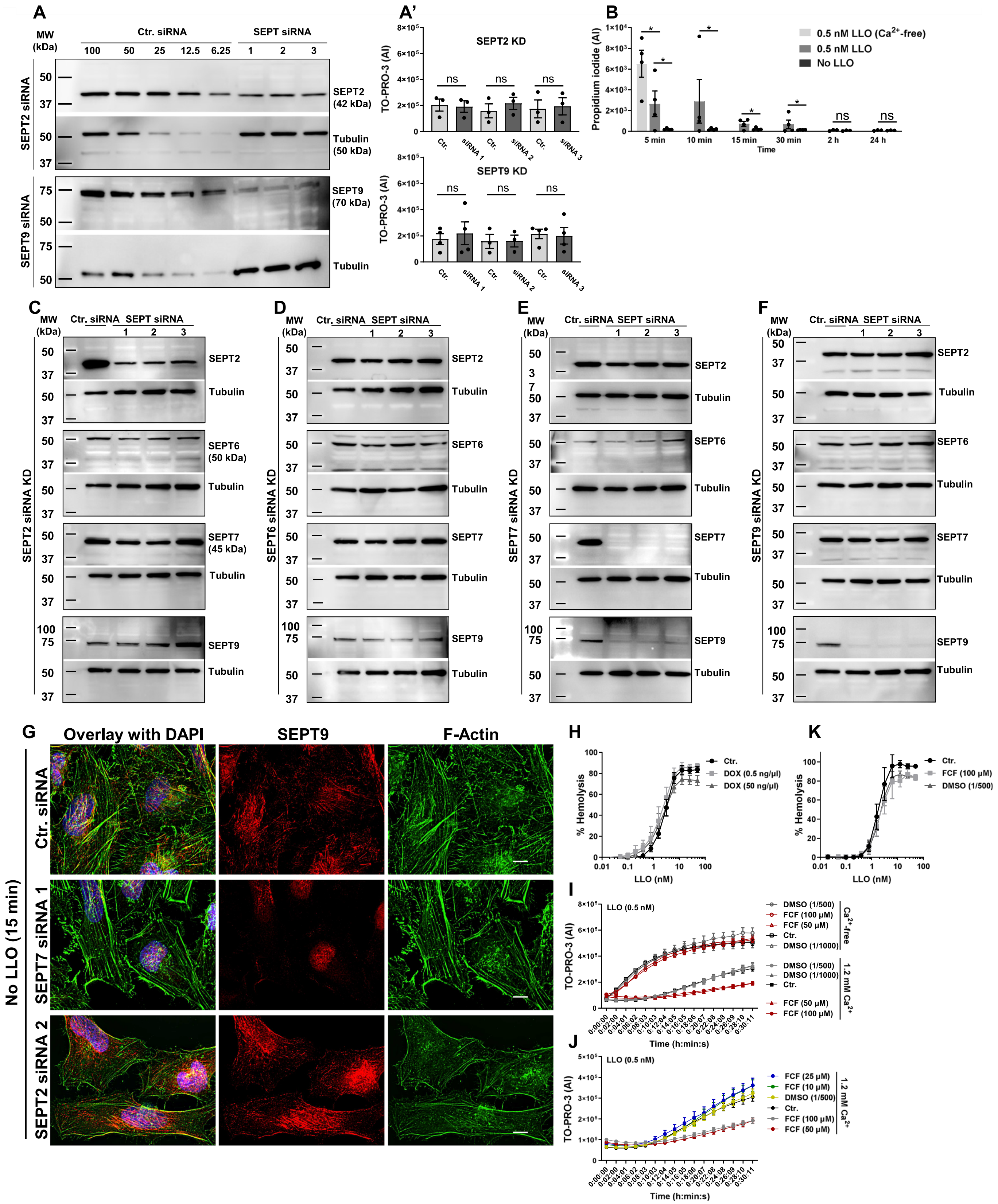
A
A'
B
Ctr. siRNA
SEPT siRNA
SEPT2 siRNA
SEPT9 siRNA
SEPT2 (42 kDa)
Tubulin (50 kDa)
SEPT9 (70 kDa)
SEPT2 KD
SEPT9 KD
TO-PRO-3 (AI)
Propidium iodide (AI)
0.5 nM LLO (Ca2+-free)
0.5 nM LLO
No LLO
Time
C
D
E
F
SEPT2 siRNA KD
SEPT6 siRNA KD
SEPT7 siRNA KD
SEPT9 siRNA KD
SEPT6 (50 kDa)
SEPT7 (45 kDa)
G
Overlay with DAPI
SEPT9
F-Actin
No LLO (15 min)
Ctr. siRNA
SEPT7 siRNA 1
SEPT2 siRNA 2
H
K
% Hemolysis
LLO (nM)
DOX (0.5 ng/µl)
DOX (50 ng/µl)
FCF (100 µM)
DMSO (1/500)
I
J
LLO (0.5 nM)
Time (h:min:s)
Ca2+-free
1.2 mM Ca2+

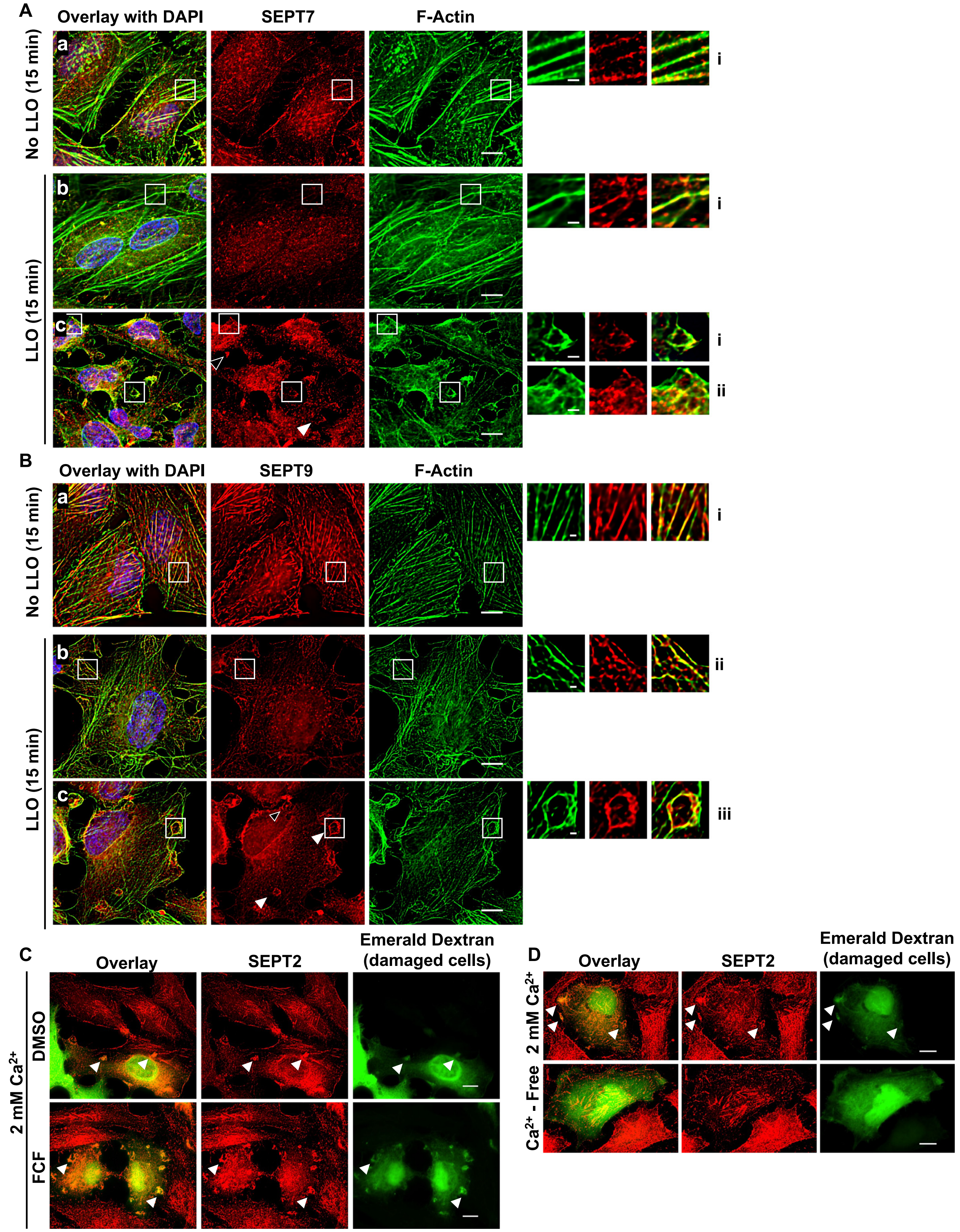
A
Overlay with DAPI
SEPT7
F-Actin
No LLO (15 min)
LLO (15 min)
a
b
c
i
i
i
ii
B
Overlay with DAPI
SEPT9
F-Actin
No LLO (15 min)
LLO (15 min)
a
b
c
i
ii
iii
C
Overlay
SEPT2
Emerald Dextran (damaged cells)
2 mM Ca2+
DMSO
FCF
D
Overlay
SEPT2
Emerald Dextran (damaged cells)
2 mM Ca2+
Ca2+ - Free

 © The Author(s)

◀ **Figure EV2. The septin cytoskeleton is remodeled in LLO-perforated and mechanically-wounded cells.**

HeLa cells were incubated without (No LLO) or with 0.5 nM LLO for 5–15 min in M1. Cells were chemically fixed, permeabilized, and fluorescently labeled with anti-SEPT9, or SEPT7, or SEPT2 primary Abs (Alexa Fluor 568-conjugated secondary), F-Actin (Alexa Fluor 488-conjugated phalloidin), and nuclei (DAPI). (**A**, **B**) SEPT7 (**A**) and SEPT9 (**B**) fluorescence images are presented with the same intensity scaling showing the loss of septin association with actin stress fibers. To better visualize septin and actin filaments, selected regions were enlarged (**A**ai, **A**bi, **A**ci,ii, **B**ai, **B**bii, **B**ciii) and the septin fluorescence display was the same for all images except for Abi and Bbii which intensity was amplified. All images were acquired by z-stack widefield microscopy, deconvolved, and presented as the best focus images, except for **A**a, **A**b, **B**a, and **B**b images which are single planes focused on actin stress fibers. Representative septin knob and loop structures are indicated by unfilled and filled arrowheads, respectively. Scale bars are 10 µm and 2 µm in the enlarged images (**C**, **D**) HeLa cells, pre-treated with 100 µM FCF, with vehicle DMSO (**C**) or untreated (**D**), were mechanically wounded in M1 (2 mM Ca^{2+}) or M2 (Ca^{2+}- Free) medium in the presence of Emerald Dextran to identify the sites of cell wounding. Cells were washed, fixed, permeabilized and fluorescently labeled with anti-septin primary Abs (Alexa Fluor 647-conjugated secondary), and nuclei (DAPI). All images were acquired by z-stacks widefield microscopy, denoised, deconvolved, and presented as the best focus images. Scale bars are 10 µm. Filled arrowheads point to remodeled septin structures.

© The Author(s)

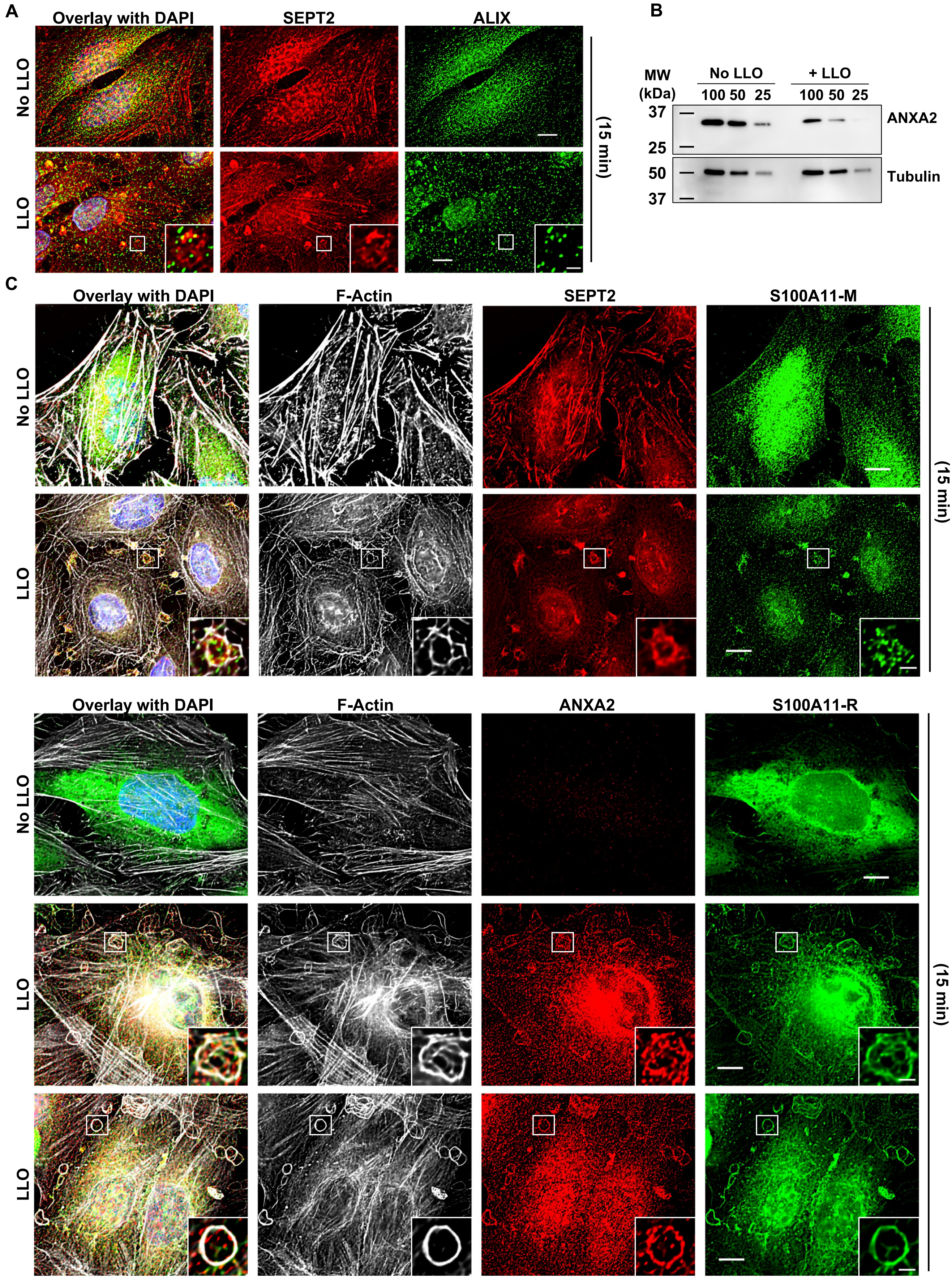
A
Overlay with DAPI
SEPT2
ALIX
No LLO
LLO
(15 min)
B
MW (kDa)
No LLO
+ LLO
100 50 25
100 50 25
37
25
50
37
ANXA2
Tubulin
C
Overlay with DAPI
F-Actin
SEPT2
S100A11-M
No LLO
LLO
(15 min)
Overlay with DAPI
F-Actin
ANXA2
S100A11-R
No LLO
LLO
LLO
(15 min)

 © The Author(s)

Figure EV3. The septin cytoskeleton redistributes with S100A11.

(**A**) Cells were fluorescently labeled for SEPT2 (Alexa Fluor 568-conjugated secondary), ALIX (Alexa Fluor 488-conjugated secondary), and nuclei (DAPI) at time point 15 min. (**B**) HeLa cells were exposed, or not, to 0.5 nM LLO for 15 min in M1. Cells were lysed and analyzed by SDS-PAGE and immunoblotting to measure ANXA2 expression level. Serial dilutions (100% to 25%) of cell lysates were loaded in the gel (representative of $N = 3$ independent experiments) and tubulin was used as loading control. Data showed that ANXA2 expression was not increased under LLO treatment. (**C**) HeLa cells were fluorescently labeled for SEPT2 (Alexa Fluor 568-conjugated secondary), F-Actin (Alexa Fluor 488-conjugated phalloidin), ANXA2 (Alexa Fluor 647-conjugated secondary), S100A11 (either anti-mouse (-M) Alexa Fluor 647-conjugated secondary or anti-rabbit (-R) Alexa Fluor 568-conjugated secondary) and nuclei (DAPI) at time point 15 min. Data Information: In (**A** and **C**), all images were acquired by z-stacks widefield microscopy, denoised, deconvolved, and presented as the best focus images except (**A**) which are single planes. Scale bars are 10 µm or 2 µm in zoomed-in regions.

© The Author(s)

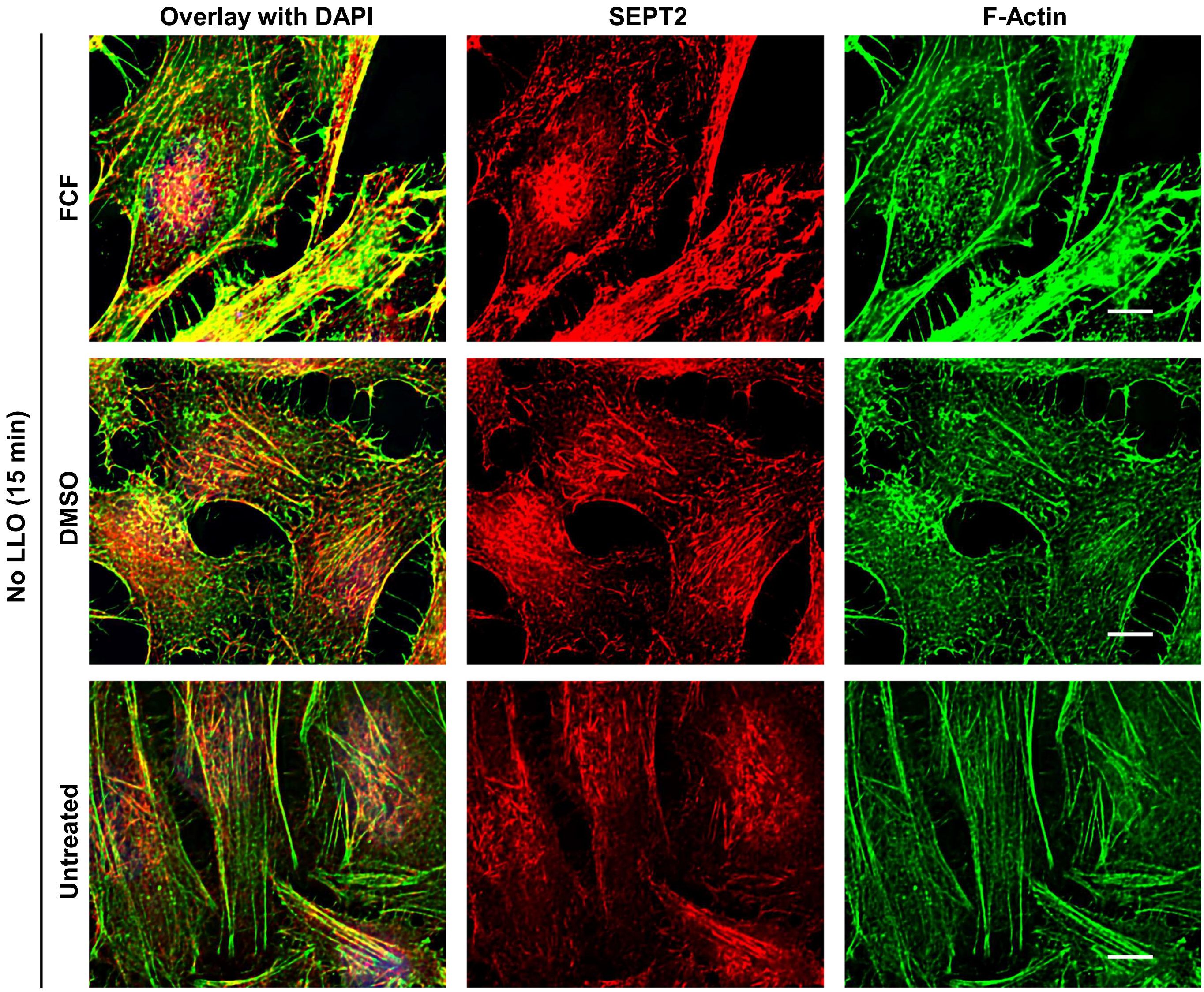

Figure EV4. Organization of the septin and actin cytoskeletons in FCF- and DMSO-pre-treated control cells.

Untreated, FCF (100 μM) pre-treated, and vehicle DMSO pre-treated HeLa cells were incubated without LLO (No LLO) for 15 min in M1. Cells were fixed, permeabilized, and fluorescently labeled for SEPT2 (Alexa Fluor 568-conjugated secondary Abs), F-actin (Alexa Fluor 647-conjugated phalloidin), and nuclei (DAPI). Z-stack images were acquired by widefield microscopy and were denoised, deconvolved, and presented as best-focus projection images. Scale bars are 10 μm.

 © The Author(s)

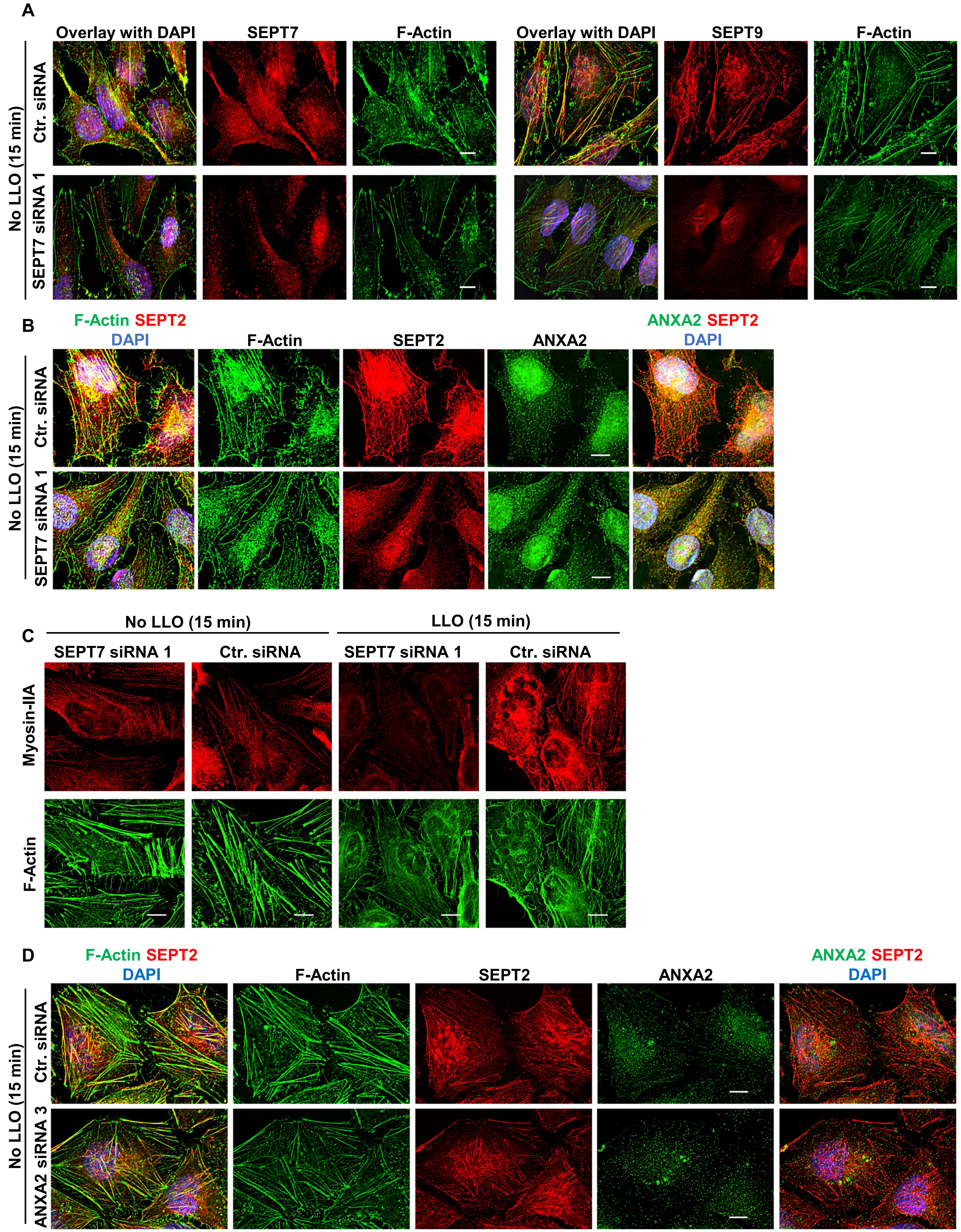
A
Overlay with DAPI
SEPT7
F-Actin
Overlay with DAPI
SEPT9
F-Actin
No LLO (15 min)
Ctr. siRNA
SEPT7 siRNA 1
B
F-Actin SEPT2 DAPI
F-Actin
SEPT2
ANXA2
ANXA2 SEPT2 DAPI
No LLO (15 min)
Ctr. siRNA
SEPT7 siRNA 1
C
No LLO (15 min)
LLO (15 min)
SEPT7 siRNA 1
Ctr. siRNA
SEPT7 siRNA 1
Ctr. siRNA
Myosin-IIA
F-Actin
D
F-Actin SEPT2 DAPI
F-Actin
SEPT2
ANXA2
ANXA2 SEPT2 DAPI
No LLO (15 min)
Ctr. siRNA
ANXA2 siRNA 3

© The Author(s)

Figure EV5. Myosin-IIA redistribution is SEPT7-dependent.

(**A–D**) HeLa cells were transfected with Ctr. siRNA, SEPT7-siRNA 1 (**A–C**), or ANXA2-siRNA 3 (**D**) for 72 h. Cells were incubated with (**C**) or without (**A–D**) LLO for 15 min in M1. Cells were fixed, permeabilized, and labeled with SEPT Ab (Alexa Fluor 568-conjugated secondary), F-Actin (Alexa Fluor 488-conjugated phalloidin), nuclei (DAPI), (**B**, **D**) ANXA2 (Alexa Fluor 647-conjugated secondary) and (**C**) Myosin IIA (Alexa Fluor 568-conjugated secondary). Scale bars: 10 µm. Data Information: (**B–D**) Z-stack images were acquired by widefield microscopy, denoised, deconvolved, and displayed as best-focus projection images, except for (**A**) which are single-plane images focused on actin stress fibers.

 © The Author(s)
